# Taking a Pause: Co‐Designing a Reflection Tool for Meaning Creation in Patients With Type 2 Diabetes

**DOI:** 10.1111/hex.70182

**Published:** 2025-02-17

**Authors:** Hyeryoung Kim

**Affiliations:** ^1^ Department of Data Science The Catholic University of Korea Seoul South Korea

**Keywords:** meaning‐creation, reflection‐sharing, self‐management, self‐reflection, type 2 diabetes

## Abstract

**Objectives:**

This study aims to explore how the design of a self‐reflection tool can assist patients with type 2 diabetes (T2D) by facilitating and sustaining their reflective practices in the context of diabetes care. Specifically, the study seeks to examine how patients utilize self‐reflection tools, identify aspects they feel improve diabetes management, and uncover the opportunities and challenges they encounter when integrating such tools into their daily lives.

**Methods:**

The research involved two phases: first, the preliminary development of a self‐reflection tool prototype and second, the exploration of user experience. During the second phase, five patients with T2D participated in three interview sessions bi‐weekly over the period of 6 weeks. The interviews were transcribed and thematically coded, and affinity diagramming was then used to synthesize the data.

**Results:**

Four main themes emerged from the interviews. The designed self‐reflection tool was found to have the potential to enhance participants’ motivation for engaging in diabetes management by improving diabetes management, initiating self‐reflection, facilitating new thoughts and meanings, and providing opportunities for the patients to share their reflections.

**Conclusions:**

The designed self‐reflection tool helped patients with T2D by encouraging them to adopt a more positive mindset and supporting them in addressing challenges related to their diabetes management. The study suggests that there is promising potential for the self‐reflection tool to evolve into a reflection‐sharing tool that can be shared with other patients.

**Patient or Public Contribution:**

Participants with T2D took part in the designed interviews, and their experiences and comments meaningfully contributed to enriching the co‐design of the self‐reflection tool. The suggested potential for this tool to be expanded into a reflection‐sharing tool is also significant.

## 1 Introduction

Type 2 diabetes (T2D) is becoming increasingly prevalent worldwide. It is reported that 6.28% of the world's population has diabetes and 38.0% of the population has prediabetes, a condition that, if not treated, often leads to T2D within 5 years [1]. One of the challenging aspects of the disease is that it is a life‐long, 24 h‐a‐day disease and the outcome of its treatment is mostly determined by patients’ health behaviours including a dietary regimen, engagement in consistent physical activity, and maintenance of an optimal blood glucose level [2]. This makes self‐management the cornerstone of diabetes care, and patients themselves need to play a crucial role in managing their illness [3, 4]. However, it is difficult for many patients with T2D to practise self‐management consistently. For example, the 2016 National Health and Nutrition Examination Survey [5] data in the United States shows that 45% of patients with diabetes have not achieved the target range of haemoglobin A1C levels, a blood test showing the average blood sugar levels. One major barrier to maintaining the optimal range of the average blood sugar levels is that self‐management of diabetes is an iterative, continuous task associated with considerable stress and distress. Patients often feel tired and overwhelmed by ongoing diabetes management, and this is known as diabetes burnout [6] or treatment fatigue [7]. For this reason, it has been well recognized in the literature [8] that strengthening and sustaining diabetic patients’ motivation is the key to successful diabetes management.

Self‐determination theory offers an insightful exploration of diabetic patients’ motivation. It is a framework of human motivation utilized across various domains, including healthcare, education, work, and sport [9]. According to this theory, people are autonomously motivated when they value specific activities for their own reasons and when they have a sense of enjoyment and interest in them. By contrast, controlled motivation involves people behaving in a certain way to avoid shame or guilt [10], which hinders their ability to effectively manage their health [11, 12, 13]. This theory is also held concerning personal meaning. A study by Deci, Eghrari, Patrick, and Leone [14] shows that individuals, on their own, tend to be more autonomously motivated to develop the necessary skills and ability to regulate their behaviours when they see those behaviours as personally meaningful. The importance of autonomously motivated behaviour is also supported in the context of diabetes management. Studies suggests that perceptions of autonomy underlie effective diabetes self‐management and thus can result in better glycemic control [15], and this autonomous motivation is developed when patients feel they are facilitating and seeing the value in specific diabetes self‐management behaviours [16, 17].

One of the critical elements for enhancing autonomous motivation in diabetes self‐management is reflection. Reflection and reflective practice are considered a close examination of one's thoughts and behaviours, learning from experience, and fostering ongoing engagement with activities [18]. It is suggested that an act of expression on experience through reflection can help patients with diabetes develop the skills necessary for adequate self‐management of diabetes [19]. One important aspect of reflection is the creation of new understanding. Gadamer [20] argues that new understanding is initiated and facilitated through reflection, and this happens through the integration of new experiences and previous understanding. In a healthcare setting, a study by Hörnsten et al. [21] suggests that reflection can contribute to increasing the awareness of patients and help them take responsibilities for their health management. In the study, learning through reflection occurred when the participating patients integrated the illnesses and change in their body with a new understanding of seeing themselves as an individual. In this process, written expression proved to be a particularly effective method for fostering such reflective learning. It is suggested that expressing individual experiences related to diabetes management can empower patients to become more self‐determined and this, in return, helps them develop the self‐care skills needed to manage diabetes [19]. The reason is that written reflection enables individuals to translate their emotions and experiences into words, so they can be more aware of the different types of situations they are facing while going through the cognitive process [22].

Given the significant relationship between self‐management in T2D, autonomous motivation, and self‐reflection, this study aims to explore how the design of self‐reflection tools can help patients with T2D by initiating and maintaining their reflection in the context of diabetes care, and how this reflection could affect their motivation and thus improve their health behaviours.

## 2 Methods

The research involved two phases: first, the preliminary work on prototype development of a self‐reflection tool, and second, the subsequent exploration of user experience. Table 1 below illustrates the research design utilized in each phase. The entire study was reviewed and approved by the Institutional Review Board (IRB) at the author's affiliated institution and was deemed exempt from full IRB review due to its minimal risk (ref. number HUM 00155695).

**Table 1:** Research design.

Phases		Activities	Methods	Participants
1	Preliminary work on prototype development of a self‐reflection tool	Applicability exploration	Interviews	13 experts 5 patients
		Prototype refinement	Informal conversations	12 participants of diabetes education class 3 diabetes educators 13 participants of diabetes support group
2	Exploration of user experience		Serial interviews	5 patients * 3 times each

### 2.1 Phase 1: Preliminary Work on Prototype Development of a Self‐Reflection Tool

**Figure 1:**
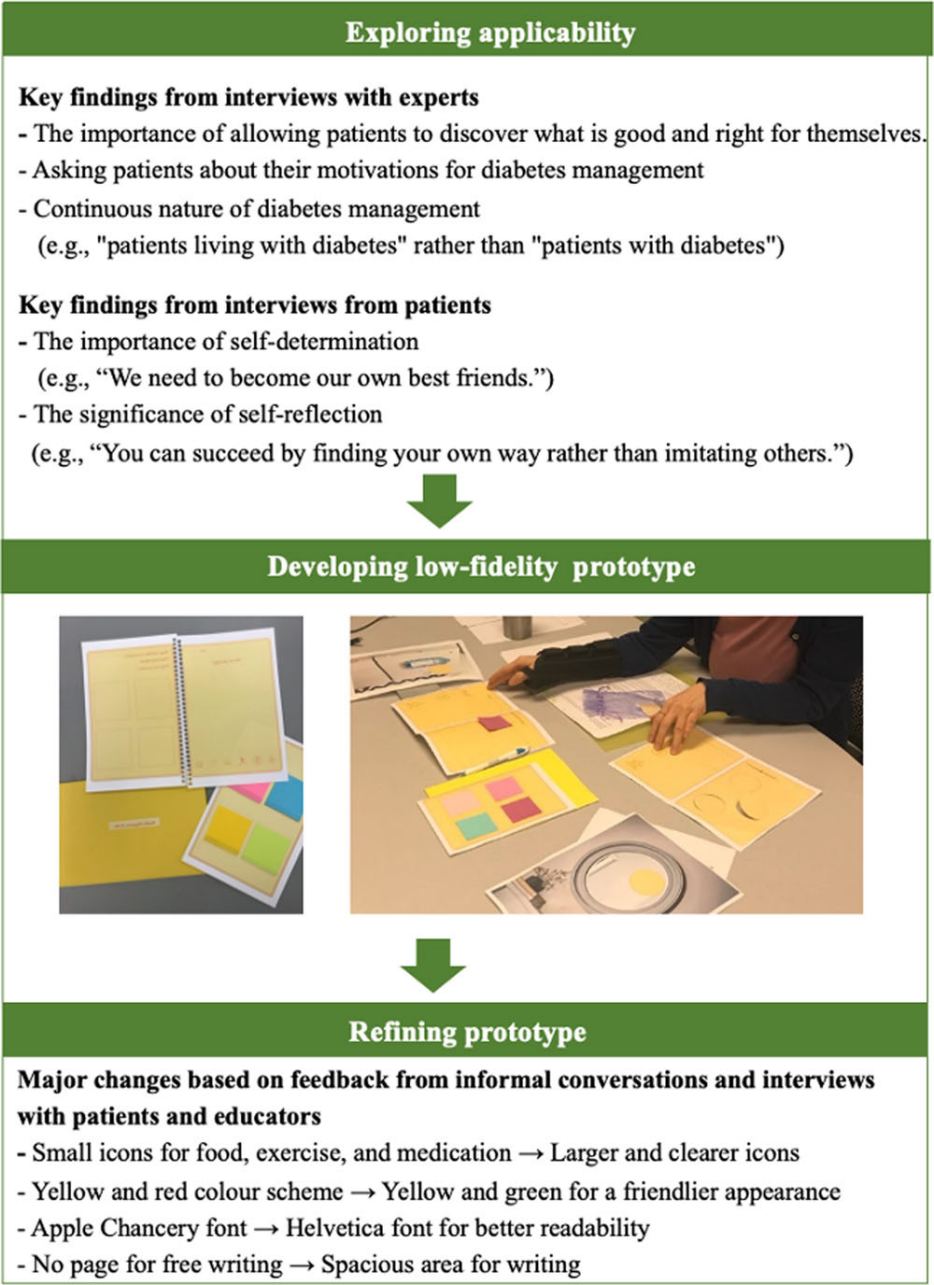
Key findings of the preliminary work in Phase 1.

In this phase, the applicability of self‐reflection was explored through interviews with subject matter experts and patients. Subject matter interviews are valuable sources when researchers need to acquire knowledge in disciplinary fields other than their own [23]. I conducted interviews with 13 experts: 2 in diabetes management, 4 in emotional management of diabetes, 2 in positive design, 4 in gratitude and mindfulness intervention and 1 in diabetes education. Also, to deepen the understanding of the contexts related to diabetes self‐management and to explore the potential for implementing a self‐reflection tool for patients with T2D, interviews were conducted with 5 patients. Patients were recruited through the Office of Patient Experience at Michigan Medicine. Subsequently, a low‐fidelity prototype of the self‐reflection tool was developed and refined through iterative adjustments based on informal verbal feedback from educators and patient participants: 12 patients and 3 diabetes educators participated from two diabetes education class sessions, as well as additional 13 patients from a diabetes support group session. Permission to attend the education class was obtained through an educational manager at the hospital, and permission to attend the diabetes support group was obtained from the nurse leader of the group. Participation was entirely voluntary. Key findings from Phase 1 are summarized in Figure 1, based on which Phase 2 was conducted.

**Figure 2:**
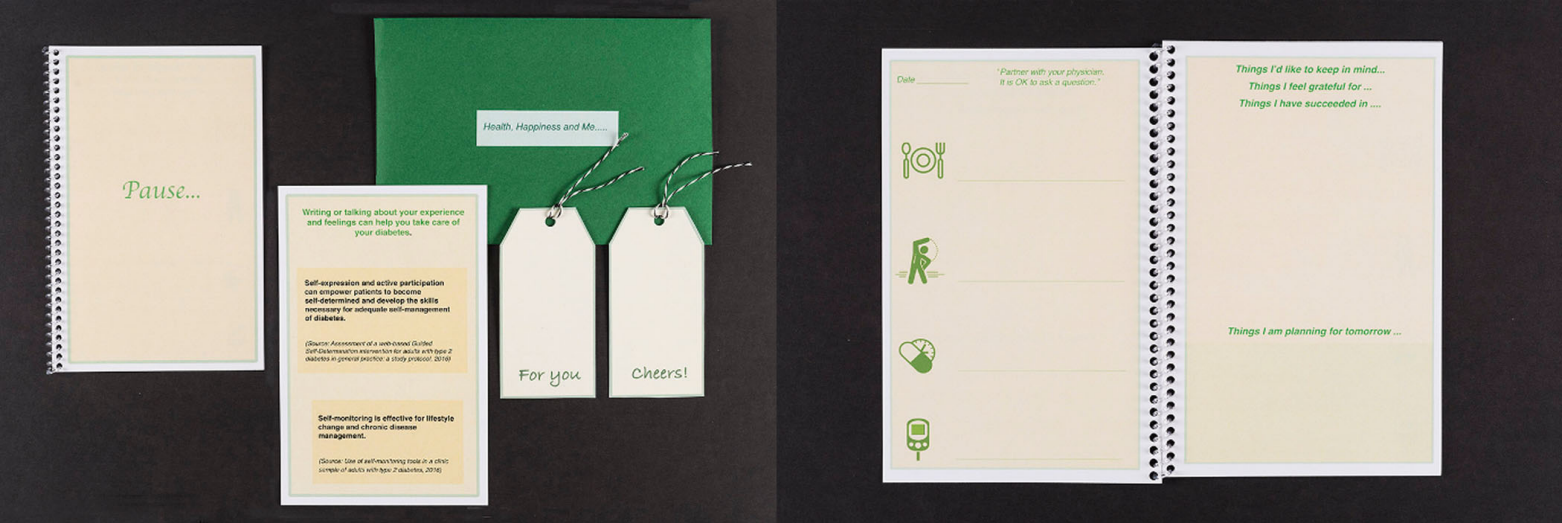
Finalized prototype.

The finalized prototype of the self‐reflection tool was a rectangle form of a packet, 8.5″ × 5.5″ in size with three components: a self‐reflection journal, a reminder, and an information card (see Figure 2). The main component is the self‐reflection journal named ‘Pause’. This implies that patients can pause at any time during a busy day and reflect on anything that has happened. The reminder component was composed of different types of materials such as sticky‐notes in different colours and paper tags which contain encouraging words such as ‘Cheers’ and ‘For you’. They were intended for the participants to write something they want to be reminded of (e.g., something they feel grateful for the day or a particular success they achieved in terms of diabetes management) and can be put to any place at home or in their workplace where they can easily see. The information card component includes positive effects of self‐reflection on diabetes care and the importance of emotional care. This was designed to encourage and convince users to participate in reflective activities.

### 2.2 Phase 2: Exploration of User Experiences

In this second phase, five patients participated, and three semi‐structured interviews with each patient were conducted over a 6‐week period. Participants were selected based on the criteria of having T2D, being willing to use intervention of a self‐reflection tool to assist in diabetes management and share their experiences, and their ability to commit to participating in three interviews. Patients were recruited through the Office of Patient Experience at the same hospital as in the first phase. It regularly recruits volunteer patients who have received care at this institution and their primary roles include providing feedback through participation in surveys or interviews conducted by the hospital or its partner hospitals. The sample size was determined based on previous usability studies that demonstrated testing with five users allows researchers to uncover 80% of user experience issues [24, 25]. Additionally, as this study aimed to explore patients’ reflective journeys into the complexities of managing diabetes as well as the usability of a self‐reflection tool, an exploratory approach involving three serial interviews was employed. Serial interviews have been shown to help researchers establish a deeper sense of familiarity and trust with participants, leading to greater participant commitment to the research process [26], and reduce biases associated with single interviews [27].

In the first interview, the researcher introduced the self‐reflection tool and how to use it, and the patients commented on it by giving their overall impression of the tool. They were told that there is no one right way to use the tool and they can be as flexible and creative as possible in using it. After the first introductory interview session, the participants took this tool home and used it for about 2 weeks.

During the second and third interviews, the main topics covered included: the circumstances of using the reflection tool, such as the time, place, and frequency and general usability; the usefulness of the reflection tool for diabetes self‐management, including the reasons behind its effectiveness or lack thereof; any unexpected or surprising experiences while using the tool; and changes in diabetes‐related behaviours, thoughts, or emotions. Each interview began with an open‐ended priming question: ‘How would you describe your experience with the self‐reflection tool?’ This allowed participants to freely share their experiences in their own words. Follow‐up probe questions were employed to clarify responses and gather more detailed information, particularly regarding specific ways in which the designed tool supported diabetes management (e.g., ‘Can you explain further?’ or ‘Could you provide more details?’).

The interviews were conducted in the studio on the author's affiliated university campus, and each interview lasted approximately 45 min to an hour. Due to the participants’ circumstances, such as having to travel to campus, the intervals between some interviews extended beyond the intended 2 weeks (see Table 2). All interviews were audio recorded on the researcher's smartphone. Verbal informed consent was obtained from the participants during the first interview after providing them with an information sheet and explaining the purpose of the study, the procedures involved, and the assurance of confidentiality regarding their responses.

**Table 2:** Participants profiles and interview dates.

Participants	Age	Duration of T2D (years)	Interval between 1st and 2nd Interviews	Interval between 2nd and 3rd Interviews
P1	74	13	13 days	14 days
P2	75	13	18 days	20 days
P3	36	5	14 days	12 days
P4	72	19	15 days	14 days
P5	57	12	15 days	13 days

### 2.3 Analysis of Data From Serial Interviews

**Figure 3:**
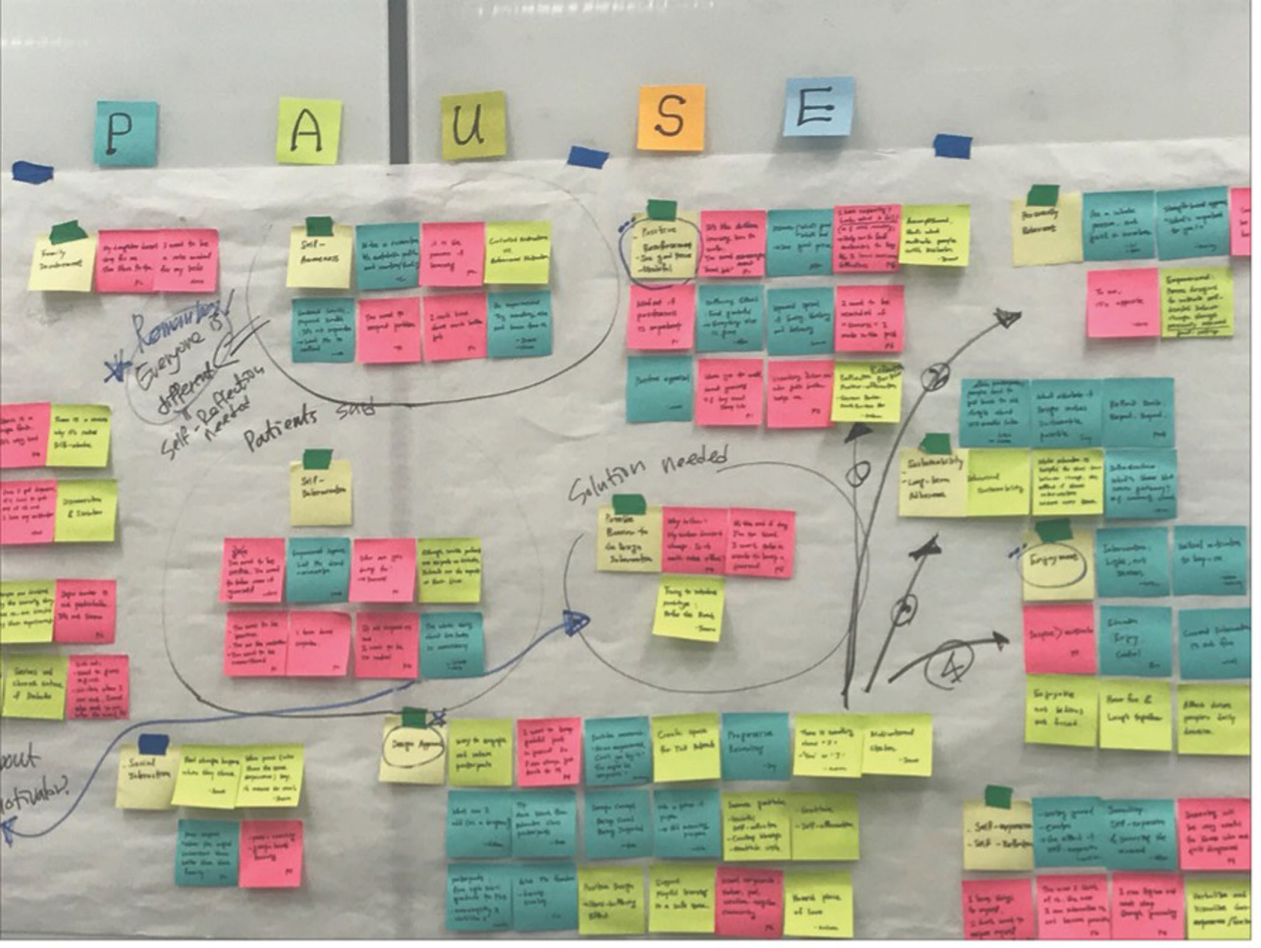
Affinity diagram.

The recorded interview sessions with the participants were transcribed and then repeatedly revisited to extract quotes relevant to the experience of using the self‐reflection tool. The quotes from the interview transcripts were initially posted randomly on a wall using sticky notes. They were then grouped into seemingly related categories, and each category was labelled (see Figure 3). This process of creating affinity diagram is a spatial clustering technique where analysts manually move around and group individual data items based on their similarity or relevance to a shared topic [28]. It has been applied for a wide variety of tasks in different domains, such as human–computer interaction, anthropology, and management [28, 29, 30] and is considered to be one of the most suitable tools for usability study [31, 32, 33]. To validate affinity diagramming [28], the affinity diagram was regularly consulted with three experts over the course of the development: a manager from the Office of Patient Experience and a diabetes specialist physician at the hospital, and a professor specializing in graphic design who regularly provided advice on this research project. These consultations prompted changes in the categorization and labelling over time. For instance, quotes initially grouped under ‘cultivating a positive mindset’ were re‐labelled as ‘facilitating new thoughts and meanings’.

## 3 Results

The analysed data from serial interviews revealed four main categories: improving diabetes management, initiating self‐reflection, facilitating new thoughts and meaning, and finding opportunities for reflection‐sharing as presented below. Table 3 outlines the main themes and corresponding quotes from participating patients.

**Table 3:** Summary of results.

Theme	Key findings	Exemplar quotes
Improving diabetes management	Increasing problem‐solving skills Planning and preparing Customizing the self‐reflection tool	‘I thought about why…. It was a discovery for me. I haven't forgot to take morning meds since then’. ‘I realized that I didn't take any exercise. So, I just stood up and stepped up and down five times before going to bed’. ‘I put a sticky note on the doorknob that says Don't go through the red light. Make good choices’.
Initiating self‐reflection	Providing an opportunity to express thoughts and feelings Responding favourably to the colour and size	‘It is a very uplifting color, and it makes me feel like trying’. ‘It was really good to sit and think about what was good and what wasn't good’. ‘I actually can write down my blood glucose numbers without feeling judged by others’.
Facilitating new thoughts and meanings	Having a more positive attitude toward life Holding a different meaning for different patients.	‘It just struck me…right… I just need to keep going with my diabetes’. ‘It will become my own memory book’. ‘I'd like to bring it with me when I see my doctor next time’.

### 3.1 Improving Diabetes Management

The participants generally found the self‐reflection tool, ‘Pause’, helpful in increasing their problem‐solving skills in their diabetes management. One patient commented on how engaging in reflection helped him in thinking about the problems he had in his diabetes management, promoting an idea, and triggering him to put into action. He stated:

One day, I realized that I forgot to take meds in the morning. I usually don't forget to take evening meds since I take other medication as well… I thought about why… what about putting the meds on the stove in the kitchen?… So, when I get up in the morning and go into the kitchen, I can instantly see the med on the stove. It was a discovery for me. I haven't forgot to take morning meds since then.

He expressed his joy of solving this long‐held problem with his idea, carrying out his idea, and being successful. Another patient commented on how reflection helped her in being more aware of the importance of doing exercise. She commented, ‘One day, I reflected on the day and realized that I didn't take any exercise. So, I just stood up and stepped up and down five times before going to bed’. The stories these two patients shared show that the self‐reflection tool can help increase awareness of the importance of the participants’ diabetes management and their commitment to it as the tool provides a chance to engage in reflection. Interestingly, while the self‐reflection tool was primarily designed for reflecting on past experiences, its potential usability for planning and preparation in diabetes management was also revealed. For instance, one participant shared an example of using a sticky note or paper tag from the provided packet as a reminder for diabetes management when she went out. She stated, ‘Sometimes I forget to take my meter with me and end up missing some important information. So, I put a sticky note on the doorknob that says, “Take your meter with you”. or ‘Don't go through the red light. Make good choices’. Another participant highlighted the importance of planning in diabetes care by using the ‘Things I am planning for tomorrow’ page to write down the next day's meals. She explained, ‘I want to make sure I bring the food from home tomorrow for lunch, pack something from home. So, I'm not either skipping my meal or looking around for something to eat or eating something not healthy’. These examples illustrate how participants customized the self‐reflection tool to align with their unique circumstances, leveraging it as a problem‐solving approach for effective diabetes management.

### 3.2 Initiating Self‐Reflection

General feedback on the design of the self‐reflection tool was overall positive and this contributed to the participants’ initial engagement in the tool. The participants responded favourably to the colour combination of yellow and green, the half letter size of the self‐reflection journal, simple design, and enough space for their reflection. One patient said, ‘I like the softness of the colors’. and another said, ‘It is a very uplifting color, and it makes me feel like trying’. The size of the journal also gained positive responses from the participants commenting that it can be put into their purse, on their work desk, or bed‐side table from which they have easy access to it and use. One of the patients shared her experience in keeping a log of blood glucose numbers saying, ‘It was painful to remember and write my numbers. There was no space for feeling. But this one allows much more flexibility. I can write or draw as much as I want’. Another patient also commented on the flexibility of the design stating, ‘The visuals help especially when I'm in a stressful condition. I like the freedom to express. It is not rigid’.

All of the five participants commented on the usefulness of the self‐reflection tool as it provides an opportunity to express their thoughts, feelings, and experiences. One participant said, ‘Rather than just go go… and do do…, it was really good to sit and just think about what was good and what wasn't good’. With respect to blood glucose numbers, a participant stated, ‘You know, I actually can write down my blood glucose numbers in this without feeling judged by others because this journal is private and it's all mine’. It is also worth noting that one of the patients commented on possible broader use of the self‐reflection tool saying, ‘This tool can be used for anybody who makes a dollar or who makes a million. Because thoughts are free!’ All of the comments above demonstrate the value of expressing one's thoughts and feelings regardless of the relevance to diabetes care. Participants’ responses were not always positive, however. One patient mentioned that her motivation to use the self‐reflection journal declined over time as it felt repetitive and monotonous. She also expressed frustration about not being able to transfer or print the journal content using her phone. She suggested that the reflection tool might be more suitable for older individuals, such as her father‐in‐law who also has diabetes, as they may find handwriting more convenient than using a mobile device. This prompted the researcher to reflect more critically on the potential influence of being both the designer of the tool and the interviewer, considering that this dual role might have led other participants to provide overly favourable responses.

### 3.3 Facilitating New Thoughts and Meanings

All five participants mentioned that a feeling of gratefulness helped them have a more positive attitude toward their life. In most cases, they associated the feeling of being grateful with their family members, friends, and daily activities rather than with diabetes‐related management. For instance, one patient said, ‘I'm grateful for having healthy kids’. Another patient also said, ‘I'm grateful to be able to give back at the bowling fundraising event for the youth’. Two of the participants particularly commented on how the self‐reflection tool helped them have new views about their life with diabetes and how those changes influenced their daily lives. One patient shared her reflection and mentioned that she needs to change one thing at a time. Another patient shared a discovery she found while watching an animated movie, Finding Nemo, with her children. She stated:

One day, I was just watching the movie, Finding Nemo, with my kids. When Nemo says ‘keep swimming, keep swimming’, it just struck me. I thought, right… I just need to keep going and keep going with my diabetes.

Both participants acquired a new understanding of and perspective on their life and their diabetes management. With regard to a broader aspect of its use, two of the patients particularly commented on how they associate with the self‐reflection tool. One patient commented, ‘I can look back on not only a certain day but also the past week and past month, and it will become my own memory book’. Another patient stated that she wanted to use the self‐reflection tool in the way of providing a better understanding for her doctor. She said, ‘I'd like to bring it with me when I see my doctor next time. I can show this tool to my doctor and say… on this day, I had pasta for lunch and I felt something’. This implies that the self‐reflection tool can hold a different meaning for different patients.

## 4 Discussion

### 4.1 Main Findings

One major finding is that self‐reflection can have a positive impact on diabetes management. This includes providing free and private space to express feelings and experiences of diabetes patients without feeling worried or judged about any poor aspects of their diabetes management, which in effect increases their problem‐solving skills in their diabetes care. All participants commented that they appreciated the time to reflect, and two of the five participants developed their own problem‐solving strategies in their diabetes management while engaging in self‐reflection. Support for this finding is found in the literature on self‐expression and therapeutic writing, which emphasizes that expressing our thoughts, feelings, and experiences can have a positive impact on our health [34]. One of the participants became more aware of her exercise routine and showed increased commitment to her regular exercise. This finding is in accord with the findings of Hörnsten et al. [21], which showed that participants took more responsibility for their diabetes management as their awareness of its importance increased through self‐reflection.

Another important finding is that the participants in this study developed new thoughts about what they were grateful for and what was important in their lives as they refreshed their memories or reflected on the events that happened to them on a specific day. These include feeling grateful for their family members and the opportunities to spend time with their loved ones like close friends, and being able to give something back to someone by participating in a charity event. Such feelings or realization came over them while engaging in what they were usually doing, such as watching an animated movie or interacting with their family or community members. This finding is in accord with the findings of Gadamer [20], which showed that a new understanding or attitude toward our lives can be created through reflective activities as we take time to pause and allow ourselves to see what is important and meaningful in our lives. All these findings underscore the importance of reflection in the context of diabetes self‐management and demonstrated how patients can be more aware of different situations they are facing through reflection and become more self‐determined in their diabetes management [22].

The findings of this study provide insights into the sustained use of self‐reflection tools. One key finding is that the self‐reflection tool can hold different meanings and associations for different patients. For some, it can serve as a personal memory book, capturing both positive and negative experiences as they live with diabetes, and for others it can become a communication tool with healthcare providers by helping them recall key events related to their diabetes management. This demonstrates that reflection tools, such as the one used in this study, can become an integral part of patients’ lives as they manage their diabetes, especially when they customize the tool and discover their own optimal ways of using it. This implies that a designed intervention for patients can be sustained over the long term when they assign personal meaning to it and gradually develop an emotional attachment to it. This aligns with findings from previous studies [35, 36, 37] that identified symbolic meaning as a critical factor in strengthening the emotional connection between users (e.g., patients) and objects (e.g., self‐reflection tools). Indeed, it is argued that meaning‐creation through self‐reflection contributes to designing sustainable solutions [38] for patients with chronic illnesses, and individuals tend to use their possessions longer when they can associate them with personal memories [39].

### 4.2 Strengths and Limitations

One notable strength of this study lies in its qualitative design, which utilized serial semi‐structured interviews, allowing participants to share their experiences with the self‐reflection tool in depth rather than conducting limited‐scale user experience testing [40]. Additionally, it is worth emphasizing that all participants completed three interviews, and a single investigator conducted all the interviews to ensure the credibility and consistency of the data collection process. The study's two‐phase exploratory design is another strength: first, obtaining feedback from stakeholders to refine the self‐reflection tool prototype, and second, conducting an in‐depth exploration of participants’ experiences. However, this study also has several limitations. First, in this study, the researcher not only designed the reflection tool but also conducted all three interviews with the participants. While this approach ensured consistency in the data collection process, it may have influenced participants’ willingness to share critical perspectives about the reflection tool, potentially leading to self‐censorship compared to interviews conducted by an independent interviewer. Moreover, the researcher's personal beliefs and values could have unintentionally shaped the interpretation of participants’ responses [41]. Reflexivity was employed to remain mindful of these dynamics and measures were taken to encouraging honest feedback, and promote the researcher's self‐critique and self‐appraisal throughout the study [42, 43, 44]. Despite these efforts, the potential for such biases remains a limitation of this study. Second, it involved participants with T2D recruited through the Office of Patient Experience, which may have introduced selection bias. As volunteer patients at the hospital, they may have been more motivated and willing to engage in reflective practices, thus limiting potential generalizability of the findings. Additionally, although five participants were considered suitable for the usability study [24, 25] and were each interviewed three times in a serial manner, the sample size remains relatively small. Previous studies [45, 46] have involved more than five participants in their usability evaluations of self‐reflection tools to obtain more comprehensive insights. Lastly, since this study primarily relied on self‐reported data without direct observation of participants’ use of the self‐reflection tool in their daily lives, it is not possible to verify the accuracy of the findings or to confirm whether participants made specific behavioural changes as a result of using the designed intervention of self‐reflection.

### 4.3 Future Studies

The findings of this study provide valuable insights for further research. First, it will be worthwhile to explore how the designed self‐reflection tool can benefit different aspects of diabetes self‐management for different patient groups. For instance, recently diagnosed patients and those who have lived with diabetes for over a decade may exhibit varying levels of knowledge about the disease and experience with self‐care. These differences necessitate further investigation into how they influence the use of self‐reflection tools or reflection‐sharing tools. Second, as T2D is a chronic condition, research that investigates ways to facilitate the long‐term adoption of self‐reflection tools, aiming to improve patient engagement in diabetes management, would be another avenue worth pursuing. As one patient noted, motivation to use self‐reflection tools may decline over time. Therefore, exploring strategies to improve the sustained use of the designed interventions is crucial [47, 48, 49]. For example, integrating self‐reflection tools with technical systems that prompt patients in real time to capture and reflect on their past successes or failures [50] could alleviate the monotony of repeated reflections, thereby providing renewed motivation for diabetes management. Lastly, research exploring the long‐term impact of self‐reflection tools on diabetes management outcomes can be meaningful. Measuring changes in patients’ HbA1C levels before and after regular, sustained use of the self‐reflection tool over an extended period could provide valuable insights into the factors influencing adherence to diabetes self‐management practices.

### 4.4 Implications for Practice

Self‐reflection tools can be incorporated into the education programs for patients with T2D in healthcare setting. By encouraging patients’ reflection, trial‐and‐error learning, and iterative progress, these tools can foster more reflective and purposeful self‐care decision‐making [51, 52]. Ultimately, such an approach can empower patients to independently discover blood glucose management strategies tailored to their individual needs—an essential component of effective diabetes self‐management. Furthermore, as noted by one of the participants in this study, the self‐reflection tool can contribute to communication between patients and physicians. Previous research has extensively documented the asymmetry in physician–patient relationships and the limitations of one‐way, hierarchical interactions [53]. For instance, a study by Holmström et al. [54] found that only 11% of diabetes care professionals focused on ensuring patients’ understanding of their condition. By addressing these gaps, self‐reflection tools have the potential to be utilized as interventions that promote two‐way, patient‐centred communication during clinical encounters. In community‐based diabetes peer support groups, leveraging self‐reflection tools could serve as a valuable resource for patients to learn from [55] and encourage one another. As demonstrated in prior research [56], shared learning can foster a supportive environment, contributing to improved self‐management outcomes.

## 5 Conclusion

Diabetes self‐management is complex, and it can be quite challenging due to the chronic and iterative nature of illness management. This study explored the potential of a self‐reflection tool to help patients with T2D to maintain their motivation in engaging in their diabetes self‐management by facilitating self‐reflection on their lives as well as illness management. The findings of this study highlight that a self‐reflection tool can enhance diabetes management, initiate and sustain self‐reflection, facilitate new perspectives on what is important and meaningful in lives, and provide opportunities for sharing reflections. This research also demonstrated that design of a self‐reflection tool can be used to identify opportunities as well as challenges related to a problem space of chronic disease like diabetes by increasing the patient involvement throughout the research process.

## Author Contributions

**Hyeryoung Kim:** conceptualization, methodology, software, data curation, investigation, validation, formal analysis, supervision, visualization, project administration, resources, writing–original draft, writing–review and editing.

## Conflicts of Interest

The author declares no conflicts of interest.

## Data Availability

The data supporting the findings of this study are available from the corresponding author upon reasonable request.
